# Analysis and Modelling of Non-Fourier Heat Behavior Using the Wavelet Finite Element Method

**DOI:** 10.3390/ma12081337

**Published:** 2019-04-24

**Authors:** Zhi-Bo Yang, Zeng-Kun Wang, Shao-Hua Tian, Xue-Feng Chen

**Affiliations:** 1 School of Mechanical Engineering, Xi’an Jiaotong University, Xi’an 710049, China; wangzengkun@stu.xjtu.edu.cn (Z.-K.W.); chenxf@mail.xjtu.edu.cn (X.-F.C.); 2 The State Key Laboratory for Manufacturing Systems Engineering, Xi’an Jiaotong University, Xi’an 710049, China

**Keywords:** non-Fourier heat conduction, film materials, Dual-Phase-Lagging, thermal behavior, wavelet

## Abstract

Non-Fourier heat behavior is an important issue for film material. The phenomenon is usually observed in some laser induced thermal responses. In this paper, the non-Fourier heat conduction problems with temperature and thermal flux relaxations are investigated based on the wavelet finite element method and solved by the central difference scheme for one- and two-dimensional media. The Cattaneo–Vernotte model and the Dual-Phase-Lagging model are used for finite element formulation, and a new wavelet finite element solving formulation is proposed to address the memory requirement problem. Compared with the current methodologies for the Cattaneo–Vernotte model and the Dual-Phase-Lagging model, the present model is a direct one which describe the thermal behavior by one equation about temperature. Compared with the wavelet method proposed by Xiang et al., the developed method can be used for arbitrary shapes. In order to address the efficient computation problems for the Dual-Phase-Lagging model, a novel iteration updating methodology is also proposed. The proposed iteration algorithms on time avoids the use the global stiffness matrix, which allows the efficient calculation for title issue. Numerical calculations have been conducted in the manner of comparisons with the classical finite element method and spectral finite element method. The comparisons from accuracy, efficiency, flexibility, and applicability validate the developed method to be an effective and alternative tool for material thermal analysis.

## 1. Introduction

The Fourier law, one of the most important laws to explain the behaviors in heat transport, seems to be ineffective in some researches when high-intense and ultra-short lasers are used as the excitation for microscale heat transports. In these experimental observations of some film materials, the sharp wave fronts responsible for temperature overshooting are hard to be interpreted by the classical model. To address this problem, some new models are proposed, like the Cattaneo–Vernotte (CV) model and the Dual-Phase-Lagging (DPL) model. The macroscopic thermal wave model was firstly postulated by Cattaneo and Vernotte in 1958. The modification leads to a hyperbolic heat condition equation and suggests describing the heat transport by wave with finite speed. However, the two-step model and the pure phonon field model proposed later suggest that the microscale thermal behavior neither follow the pattern given by the CV model nor Fourier diffusion model. To fill the gap between microscopic to macroscopic theories, the DPL model was proposed by Joseph [1] and Tzou [2,3] according to two time constants in the thermal evolution equation. The DPL model aims to remove the precedence assumption made in the CV model. It allows either the temperature gradient to precede the heat flux vector or the heat flux vector to precede the temperature gradient in the transient process [4]. The model tries to lump the microstructural effect into the delay of response in time. Although it is still a postulated model, some experimental observations given by Tzou and Tang et al. [5,6] have shown the well agreement with it. It should be emphasized that the CV and DPL model used in many works are derived using a Taylor series expansion. In fact, this way of derivation is not compatible with the second law of thermodynamics. Moreover, the DPL equation is a special, linearized version of the Jeffrey equation which is compatible with thermodynamics. In Rukolaine’s work [7,8,9,10], it is found that the parameters appearing in the DPL equation are not independent from each other. Thus, they cannot be arbitrary, otherwise the solutions can be unphysical.

Compared with the parabolic diffusion equation of Fourier model, the CV and DPL models are hyperbolic in nature. As a result, there is a resurgent interest in the solution of the heat conduction equations given by the CV or DPL model, which accounts for the finite propagation velocity of a thermal wave within the inspected media A survey of numerical schemes for the solving of the heat conduction equation can be organized into two categories, namely analytical solutions [4,11] and numerical solutions [12,13,14,15]. Some remarkable analytical methodologies including the Laplace transformation [16], Green’s function [17], and the integral equation method [18] have been widely investigated. The details of these analytical solution methodologies can be referred to Wang’s book [11] for more information. Without doubt, the accuracy and efficiency of analytical solution are unparalleled compared to these properties of numerical methods. However, we cannot overlook the sophisticated mathematical skills and the complex transformation required in analytical methods, which have been the main obstacles for their application in practice. Due to the complexity of hyperbolic equation, especially the complex inspected region, only very few simple cases can be solved analytically. Consequently, more attentions have been drawn by the numerical solutions and methodologies. However, the accurate solution for film material and two-dimensional medias are sometimes not easily obtained [19,20]. The finite difference method appears to be firstly used to analyze this problem by Yeung et al. [21,22]. They introduced a simple and concise finite difference algorithm developed by applying the Godunov scheme on the characteristic equation. After that, Han and Tang et al. extended the work to the two-dimensional media [23]. Dai et al. further developed the finite difference algorithm, based on which they proposed the convergent three-level finite difference scheme [24] and a high order accurate finite difference method for solving the two-dimensional DPL problem [25]. Boltzmann method is also a popular method used to solve the title problem. Wang et al. [26] developed an enhanced Gray model by considering the second-order terms in Taylor expansion in the phonon Boltzmann transport equation. Xu and Wang derived the DPL model using the Boltzmann method, and investigated the oscillation of the microscale heat conduction systemically [27,28]. Beside the above methods, the classical finite element method (FEM) is also usually used. Ai et al. [29,30] constructed a discontinuous FEM model and analyzed the thermal wave propagation in one- and two-dimensional medias using DPL model. Motivated by these works, this article aims to develop the wavelet finite element (WFEM) formulation and the corresponding solving methodologies for non-Fourier heat conduction. The wavelet finite element is a novel element proposed by Xiang et al. [31,32]. In the authors’ former works [33,34,35,36], the effectiveness of WFEM has been verified for dynamic analysis and elastic wave problems. However, the difference between former issues with the thermal problem make it necessary to develop the WFEM. Now the WFEM is seldomly used in thermal analysis, the only available works are developed by Zhao et al. [37]. Zhao’s works show the potential of WFEM for thermal analysis. Considering the non-Fourier heat conduction problems have not been investigated, we focus on the WFEM formulation for CV and DPL models.

This paper is organized as follow: Section 2 presents the basic models used in this work, based on that, Section 3 presents the numerical formulations for solving by WFEM. The numerical results and discussions are given in Section 4 for validation.

## 2. Problem Descriptions

### 2.1. Cattaneo–Vernotte Model (CV Model)

In classical heat conduction investigations, the diffusion of heat is characterized by the empirical law (Fourier law of heat conduction), which postulates that the heat flux is directly proportional to the temperature gradient as:

(1) q(r,t)=−k∇T(r,t)

where **q** is the heat flux, *r* is the position vector, *t* is the physical time, *k* is the conductivity for thermal medium, and *T* is the temperature. Since the diffusion equation is parabolic by nature, it is easy to know from the wave motion view that Equation (1) implies an infinite speed of the propagation of thermal wave, which indicates that the local change in heat flux **q** can lead to an instantaneous perturbation in temperature field *T*. It has been verified that the conclusion is incompatible with experiments. Based on the two-fluid model, Tisza [38] predicted the existence of thermal wave, which was detected by Peshkov [39] as the “second sound”. Associated with the development of material processing via pulsed sources and the requirement of laser induced guide wave in structural health monitoring, the classical Fourier’s law was shown to be inadequate in modelling the high frequency response. The problems mentioned above triggered many attempts to improve the classical model, the most famous one appears to be the CV model, in which the thermal “inertia” is taken into account [7,8]:

(2) $$\tau_{0}\frac{\partial q\left(r,t\right)}{\partial t}+q\left(r,t\right)=-k\nabla T\left(r,t\right)$$

Equation (2) is also the first order approximation to the single-phase-lag constitutive relation:

(3) $$q\left(r,t+\tau_{0}\right)=-k\nabla T\left(r,t\right)$$

relaxation time is defined by $\tau_{0}$. The physical meaning of $\tau_{0}$ can be interpreted as the natural result of communication time in molecules collisions in material, which is further formulated as:

(4) $$\tau_{0}=\frac{\alpha }{v_{CV}^{2}}\Leftrightarrow v_{CV}^{}=\sqrt{\frac{\alpha }{\tau_{0}}}=\sqrt{\frac{k}{\rho c\tau_{0}}}$$

where *α* = *k*/*ρc* is the thermal diffusivity, *ρ* and *c* are the density and the specific heat of the material. The introduction of relaxation time allows the time lag between heat flux and the change of temperature, and hence the thermal wave propagation can be described by this model. Equation (3) and the energy equation:

(5) $$\rho c\frac{\partial }{\partial t}T\left(r,t\right)+\nabla \cdot q\left(r,t\right)=Q\left(r,t\right)$$

where Q(r,t) depicts the heat source, yield the delay heat equation:

(6) $$\frac{\partial }{\partial t}T\left(r,t\right)-\alpha \Delta T\left(r,t-\tau_{0}\right)=Q\left(r,t\right)$$

However, the initial value problems for Equation (6) are ill-posed. Therefore, the single-phase-lag constitutive relation (Equation (3)) cannot be considered as sensible physical one [7,8]. In the present references, the Jeffreys-type constitutive relation [1] is usually used to approximate the thermal behavior:

(7) $$\tau_{0}\frac{\partial^{2}}{\partial t^{2}}T\left(r,t\right)+\frac{\partial }{\partial t}T\left(r,t\right)-\alpha \Delta T\left(r,t\right)=\frac{1}{\rho c}Q\left(r,t\right)+\frac{\tau_{0}}{\rho c}\frac{\partial }{\partial t}Q\left(r,t\right)$$

The initial value problems of Equation (7) are well-posed. It should be emphasized that the Jeffreys-type [9,10] constitutive relation Equation (7) cannot be considered as a real approximation of the single-phase-lag relation leading to ill-posed problems.

### 2.2. Dual-Phase-Lagging Model (DPL Model)

It has been confirmed by experimental data that the CV model performs better than the classical Fourier law in numerical prediction. The CV model, however, may obtain some predictions which cannot be supported by experiments. A comprehensive study shows that the CV model has only taken account of the fast-transient effects, but not the micro structural interactions. These two effects can be reasonably represented by the DPL between **q** and ∇T:

(8) $$q\left(r,t+\tau_{0}\right)=-k\nabla T\left(r,t+\tau_{T}\right)$$

where $\tau_{T}$ is the delay time caused by the micro-structural interactions such as phonon–electron interaction or phonon scattering and is called the phase-lag of the temperature gradient. Similarly, we can obtain the corresponding delay heat equation by Equations (5) and (8):

(9) $$\frac{\partial }{\partial t}T\left(r,t\right)-\alpha \Delta T\left(r,t-\tau_{0}+\tau_{T}\right)=Q\left(r,t\right)$$

Likewise, the initial value problems of Equation (9) are ill-posed. The Jeffreys-type constitutive relation of the DPL model is given by:

(10) $$\tau_{0}\frac{\partial^{2}}{\partial t^{2}}T\left(r,t\right)+\frac{\partial }{\partial t}T\left(r,t\right)-\alpha \Delta T\left(r,t\right)-\alpha \tau_{T}\frac{\partial }{\partial t}\Delta T\left(r,t\right)=\frac{1}{\rho c}Q\left(r,t\right)+\frac{\tau_{0}}{\rho c}\frac{\partial }{\partial t}Q\left(r,t\right)$$

The initial value problems of Equation (10) are also well-posed, thus the Jeffreys-type constitutive relation cannot be considered as a strict description of the DPL model. The higher-order approximations of CV and DPL model were also considered in literatures see in Prof. Rukolaine’s and Prof. Chirita’s works [7,40,41,42,43].

### 2.3. The Dimensionless Formulation

Consider that the parameters involved in calculations are very extreme, which generate the difficulty in simulations. The CV and DPL models are usually transformed into the corresponding normalized forms. Firstly, the excitation is nondimensionalized following the formulation introduced in [23]. Traditionally, the Gaussian profile is used to simulate the light intensity of laser pulses:

(11) $$Q=\frac{\left(1-R\right)I_{0}}{\sqrt{\pi }t_{p}^{}}\exp(-1-\frac{t^{2}}{t_{p}^{2}})$$

where *R* is the reflectivity of irradiated surface, *I*_0_ is the output intensity of laser, *t_p_* is the full-width-at-half-maximum of pulse. For convenience in the subsequent analysis, the following dimensionless parameters are introduced following the definition in [23]:

(12) $$\mathrm{Length}\;\mathrm{parameters}:\;X=\frac{x}{2\sqrt{\alpha \tau_{0}}},\;Y=\frac{y}{2\sqrt{\alpha \tau_{0}}},\;R_{c}=\frac{r_{c}}{2\sqrt{\alpha \tau_{0}}},$$

(13) $$\mathrm{Time}\;\mathrm{parameters}:\;\lambda =\frac{t}{2\tau_{0}},\;\lambda_{p}=\frac{t_{p}}{2\tau_{0}},\;\lambda_{T}=\frac{\tau_{T}}{2\tau_{0}},$$

(14) $$\mathrm{Temperature}\;\mathrm{parameter}:\;\Theta =\sqrt{\frac{\pi \tau_{0}}{\alpha }}\frac{k\left(T-T_{0}\right)}{\left(1-R\right)I_{0}},$$

(15) $$\mathrm{Heat}\;\mathrm{flux}\;\mathrm{parameter}:\;\varphi =\frac{\tau_{0}\sqrt{\pi }}{\left(1-R\right)I_{0}}q,$$

(16) $$\mathrm{Heat}\;\mathrm{source}\;\mathrm{parameter}:\;\psi =\frac{2\tau_{0}\sqrt{\pi \alpha \tau_{0}}}{\left(1-R\right)I_{0}}Q,$$

where *T*_0_ is the reference temperature. Based on the above dimensionless parameters, Equation (7) for the CV model is rewritten as the dimensionless form:

(17) $$\frac{\partial^{2}}{\partial \lambda^{2}}\Theta +2\frac{\partial }{\partial \lambda }\Theta -\Delta \Theta =2\psi +\frac{\partial }{\partial \lambda }\psi$$

and the dimensionless DPL model of Equation (10) is rewritten as:

(18) $$\frac{\partial^{2}}{\partial \lambda^{2}}\Theta +2\frac{\partial }{\partial \lambda }\Theta -\Delta \Theta -\lambda_{T}\frac{\partial }{\partial \lambda }\Delta \Theta =2\psi +\frac{\partial }{\partial \lambda }\psi$$

It is easy to know the CV model can be obtained by the DPL model by setting $\lambda_{T}=0$, namely $\tau_{T}=0$, in Equation (18). Thus, only the derivation of Equation (18) is presented here. Firstly, substituting the length parameters in Equation (10) by dimensionless forms (either for variables or operators), we get (here **R** is used to define the dimensionless **r**):

(19) $$\tau_{0}\frac{\partial^{2}}{\partial t^{2}}T\left(R,t\right)+\frac{\partial }{\partial t}T\left(R,t\right)-\frac{1}{4\tau_{0}}\Delta T\left(R,t\right)-\frac{\tau_{T}}{4\tau_{0}}\frac{\partial }{\partial t}\Delta T\left(R,t\right)=\frac{1}{\rho c}Q\left(R,t\right)+\frac{\tau_{0}}{\rho c}\frac{\partial }{\partial t}Q\left(R,t\right)$$

Thereafter, substituting the time parameters in Equation (19) by dimensionless forms:

(20) $$\frac{\partial^{2}}{\partial \lambda^{2}}T\left(R,\lambda \right)+2\frac{\partial }{\partial \lambda }T\left(R,\lambda \right)-\Delta T\left(R,\lambda \right)-\lambda_{T}\frac{\partial }{\partial \lambda }\Delta T\left(R,\lambda \right)=\frac{4\tau_{0}}{\rho c}Q\left(R,\lambda \right)+\frac{4\tau_{0}}{2\rho c}\frac{\partial }{\partial \lambda }Q\left(R,\lambda \right)$$

then replace the temperature parameter by dimensionless parameters, considering the relation that *α* = *k/ρc*, we get:

(21) $$\frac{\partial^{2}}{\partial \lambda^{2}}\Theta \left(R,\lambda \right)+2\frac{\partial }{\partial \lambda }\Theta \left(R,\lambda \right)-\Delta \Theta \left(R,\lambda \right)-\lambda_{T}\frac{\partial }{\partial \lambda }\Delta \Theta \left(R,\lambda \right)=\frac{4\tau_{0}\sqrt{\alpha \pi \tau_{0}}}{\left(1-R\right)I_{0}}Q\left(R,\lambda \right)+\frac{2\tau_{0}\sqrt{\alpha \pi \tau_{0}}}{\left(1-R\right)I_{0}}\frac{\partial }{\partial \lambda }Q\left(R,\lambda \right)$$

Lastly, replace the heat source parameter by dimensionless parameters:

(22) $$\frac{\partial^{2}}{\partial \lambda^{2}}\Theta \left(R,\lambda \right)+2\frac{\partial }{\partial \lambda }\Theta \left(R,\lambda \right)-\Delta \Theta \left(R,\lambda \right)-\lambda_{T}\frac{\partial }{\partial \lambda }\Delta \Theta \left(R,\lambda \right)=2\psi \left(R,\lambda \right)+\frac{\partial }{\partial \lambda }\psi \left(R,\lambda \right)$$

which is simply denoted in the form given in Equation (18). In above equations, the dimensionless heat source is given by (combine Equations (11) and (16)):

(23) $$\psi =\frac{\sqrt{\alpha \tau_{0}}}{\lambda_{p}}\exp\left(-1-\frac{\lambda^{2}}{\lambda_{p}{}^{2}}\right)$$

## 3. Numerical Model

### 3.1. Wavelet Interpolating/Shape Function

In above sections, the basic CV and DPL models are presented in the form of partial differential equations (PDE). In the past decades, many excellent approaches have been developed to obtain the close-form solution of these PDEs, the typical works are referred to Tzou’s work and Wang’s work [2,11]. The numerical methods, especially the finite element method, however, is more flexural for complex boundary conditions and modelling. The PDEs are then transformed into the formulations which can be used in the FEM.

Similar as the classical finite element method, the region Ω is firstly meshed in terms of a set of nonoverlapping sub-domain $\Omega_{e}$, and each sub-domain is mapped to a unit interval considering the dimension of the problem analyzed. In the unit interval, some *m*th-order *j* scale B-spline wavelets on interval $\phi_{m,k}^{j}(\xi )$ (BSWI), are used to construct wavelet finite element formulations for title problem. According to the *m*th-order 0 scale B-spline functions and the corresponding wavelets given by Goswami [44], the j scale *m*th-order BSWI, which is simply denoted as BSWI*mj*, can be defined. The support of the inner B-spline occupies m segments:

(24) $$\{\begin{array}{l} 0\;\mathrm{boundary}:\;x_{-m+1}^{j}=x_{-m+2}^{j}=\ldots =x_{0}^{j}=0\\ \mathrm{inner}\;\mathrm{knots}:\;x_{k}^{j}=k2^{-j}\;k=0,1,\ldots ,2^{j}\\ 1\;\mathrm{boundary}:\;x_{2^{j}+1}^{j}=x_{2^{j}+2}^{j}=\ldots =x_{2^{j}+m-1}^{j}=1 \end{array}$$

At any scale *j*, the discretization step is 1/2*^j^*. Thus, in order to have at least one inner B-spline function, the following condition should be satisfied:

(25) $$2^{j}\geq 2m-1$$

Let *j*_0_ be the initial scale, then for each $j\geq j_{0}$,

(26) $$\phi_{m,k}^{j}(\xi )=\left\{ \begin{array}{l} \phi_{m,k}^{j_{0}}(2^{j-j_{0}}\xi ),\;k=-m+1,\ldots ,-1\\ \phi_{m,2^{j}-m-k}^{j_{0}}(1-2^{j-j_{0}}\xi ),\;k=2^{j}-m+1,\ldots ,2^{j}-1\;\\ \phi_{m,0}^{j_{0}}(2^{j-j_{0}}\xi -2^{-j_{0}}k),\;k=0,\ldots ,2^{j}-m \end{array}\right.\begin{array}{l} \left(0\;\mathrm{boundary}\;\mathrm{scaling}\;\mathrm{functions}\right)\\ \left(1\;\mathrm{boundary}\;\mathrm{scaling}\;\mathrm{functions}\right)\\ \left(\mathrm{inner}\;\mathrm{scaling}\;\mathrm{functions}\right) \end{array}$$

**Figure 1:**
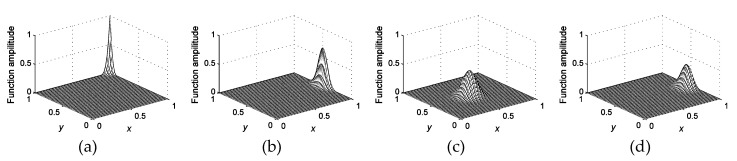
Some typical two-dimensional B-spline wavelets on interval BSWI4_3_ scaling functions: (**a**) corner function; (**b**) boundary function; (**c**) inner function; (**d**) partly inner function.

The scaling functions $\phi_{m,k}^{j}(\xi )$ (m≥2) can be derived by the following formulas [44]:

(27) $$\phi_{m,k}^{j}(\xi )=\left(x_{k+m}^{j}-x_{k}^{j}\right)\times {\left[x_{k}^{j},x_{k+1}^{j},\ldots ,x_{k+m}^{j}\right]}_{x}{\left(x-\xi \right)}_{+}^{m-1}$$

where the function ${\left(x\right)}_{+}^{}≐\max\left(0,x\right)$, and ${\left[x_{k}^{j},x_{k+1}^{j},\ldots ,x_{k+m}^{j}\right]}_{x}$ is the *m*th-order divided difference of ${\left(x-\xi \right)}_{+}^{m-1}$ with respect to *x*. Referring to Equation (26), we can derive any $\phi_{m,k}^{j}(\xi )$ from $\phi_{m,k}^{0}(\xi )$. Based on Equation (24), the BSWI4_0_ (*m* = 4, *j* = 0) functions are calculated [44], and then the expressions of BSWI4_3_ (*m* = 4, *j* = 3) scaling functions are obtained and used as the main interpolating function and shape function in WFEM for example. Restricted by space, we cannot present the BSWI4_3_ scaling functions in detail. For two-dimensional case, we further define the horizontal and vertical interpolating vectors based on Equation (27):

(28) $$\phi_{\xi }≐\left\{\phi_{m,-m+1}^{j}(\xi )\;\phi_{m,-m+2}^{j}(\xi )\ldots \phi_{m,2^{j}-1}^{j}(\xi )\right\},\phi_{\eta }≐\left\{\phi_{m,-m+1}^{j}(\eta )\;\phi_{m,-m+2}^{j}(\eta )\ldots \phi_{m,2^{j}-1}^{j}(\eta )\right\}$$

where *ξ*, *η* belong to the interval [0, 1], which depict the normalized *x* and *y* coordinates, respectively. The two-dimensional interpolating function is formulated based on the Kronecker product (⊗) between the two vectors in Equation (28), namely $\Phi =\phi_{\xi }⊗\phi_{\eta }$. Figure 1 presents some typical BSWI4_3_ functions in two-dimensional analysis.

In the frame of finite element method (FEM), the unknown continuous temperature field function T(ξ,η,t) can be interpolated in elemental region as:

(29) $$T\left(\xi ,\eta ,t\right)=NT^{e}$$

where **N** is the interpolating function, and **T***^e^* is the nodal temperature in an element. Since there are more than one node in an element, interpolating function and nodal temperature are both written in matrix form. In this work, the BSWI4_3_ function is selected as the interpolating function **N**. Since the physical filed is recorded in terms of wavelet coefficients in wavelet interpolations, an additional transform matrix $\hat{T}$ is required to transform the wavelet coefficients into the physical domain. Thereafter, the interpolating function **N** yields:

(30) $$\Phi \hat{T}=N$$

where the transform matrix is $\hat{T}={\left\{\phi_{\xi }^{T}(\xi_{1}),\;\phi_{x}^{T}(\xi_{2})\;\ldots \;\phi_{x}^{T}(\xi_{n+1})\right\}}^{-T}⊗{\left\{\phi_{\eta }^{T}(\eta_{1}),\;\phi_{y}^{T}(\eta_{2})\;\ldots \;\phi_{y}^{T}(\eta_{n+1})\right\}}^{-T}$.

### 3.2. WFEM Formulation

The PDE given for the CV model is transformed into the WFEM formula in this section by aid of the trial function. Equation (17) is the strong form of CV model, where the variables (*X*, *Y*, λ) for temperature and heat source are abbreviated for the sake of convenience. The requirement of the two-order continuity of Θ, namely the component ΔΘ, compounds the difficulty of trial function selection in WFEM. Therefore, the weak form is usually used. By dotting Equation (17) with the trial function ϑ, and integrating it by parts over the region of interest Ω, one can get:

(31) $$\int_{\Omega }\vartheta \frac{\partial^{2}}{\partial \lambda^{2}}\Theta d\Omega +2\int_{\Omega }\vartheta \frac{\partial }{\partial \lambda }\Theta d\Omega +\int_{\Omega }\nabla \vartheta \nabla \Theta d\Omega =\int_{\Omega }\vartheta \left(2\psi +\frac{\partial }{\partial \lambda }\psi \right)d\Omega$$

Based on the Hamilton’s principle, the weak form of CV model can be obtained in a matrix form, which yields:

(32) $$M\frac{\partial^{2}}{\partial \lambda^{2}}\Theta +C\frac{\partial }{\partial \lambda }\Theta +K\Theta =G$$

where the matrixes are defined by:

(33) $$M=\sum_{e}^{}\sum_{i}^{n+1}\sum_{j}^{n+1}w_{i}w_{j}N^{T}N\det\left(J\right)$$

(34) $$C=\sum_{e}^{}\sum_{i}^{n+1}\sum_{j}^{n+1}2w_{i}w_{j}N^{T}N\det\left(J\right)$$

(35) $$K=\sum_{e}^{}\sum_{i}^{n+1}\sum_{j}^{n+1}w_{i}w_{j}\nabla N^{T}\nabla N\det\left(J\right)$$

(36) $$G=\sum_{e}^{}\sum_{i}^{n+1}\sum_{j}^{n+1}w_{i}w_{j}N^{T}\left(2\psi +\frac{\partial }{\partial \lambda }\psi \right)\det\left(J\right)$$

The symbol *e* defines the total number of finite elements used in modelling, *i* and *j* are the indexes of element on different directions, *w_i_* and *w_j_* are the corresponding weights of Gaussian integrations, and matrix **J** is the Jacobi matrix. The calculation methodology for these parameters are referred to the basic theory of FEM [45], thus they are not presented in details. Since the structure of Equation (32) is same with the typical wave propagation equation or dynamic responses in elastic problems, it can be predicted that the temperature change propagates in the wave-like mode.

The same process of the CV weak form is used to generate the corresponding weak form for the DPL model, where **M**, **C**, **K,** and **G** have been defined in Equations (33)–(36):

(37) $$\int_{\Omega }\vartheta \frac{\partial^{2}}{\partial \lambda^{2}}\Theta d\Omega +2\int_{\Omega }\vartheta \frac{\partial }{\partial \lambda }\Theta d\Omega +\int_{\Omega }\nabla \vartheta \nabla \Theta d\Omega +\lambda_{T}\int_{\Omega }\nabla \vartheta \frac{\partial }{\partial \lambda }\nabla \Theta d\Omega =\int_{\Omega }\vartheta \left(2\psi +\frac{\partial }{\partial \lambda }\psi \right)d\Omega$$

(38) $$M\frac{\partial^{2}}{\partial \lambda^{2}}\Theta +\left(C+H\right)\frac{\partial }{\partial \lambda }\Theta +K\Theta =G$$

The difference between the DPL and the CV model is mainly focused on **H**, a matrix who plays the role of damping but derived from **K**. The ratio of relaxation time $\tau_{T}/\tau_{0}$ determines the properties of the DPL model:

(39) $$H=\lambda_{T}\sum_{e}^{}\sum_{i}^{n+1}\sum_{j}^{n+1}w_{i}w_{j}\nabla N^{T}\nabla N\det\left(J\right)=\lambda_{T}K$$

Equations (32) and (38) present the basic solving formulation of WFEM for the CV and DPL PDEs, however, these basic formulations are only suitable for the small-scale computation, namely the degrees of freedoms (*Dof*) are strictly restricted because of limitation of computer memory. To address this problem, a special solving methodology is proposed in this work.

### 3.3. Solving Methodology

The direct solving methodologies for Equations (32) and (38) are well-established in numerical analysis, such as the mode superposition scheme and the central difference time integration scheme. These methodologies, however, are restricted in small *Dof* issue due to the limitation of the hard memory of computer. In order to interpret this problem, we can consider 1000 *Dofs* structure to be inspected by CV or DPL model. The *Dofs* considered here is still a relatively small scale for calculation. By aid of the weak forms, the 1000 × 1000 matrixes **M**, **C**, **H,** and **K**, and the 1000 × 1 vector **G** can be obtained. Firstly, one can try to solve Equations (32) and (38) by mode superposition method. In this scheme, the inverse of a 1000 × 1000 matrix should be calculated in advance to get the 1st-1000th thermal modal shapes. Thereafter, the mode superposition can be conducted. Totally, the inverse of a 1000 × 1000 matrix cannot be directly calculated in a common computer due to the problem of efficiency, the advanced numerical method like the Lancozs method should be utilized. It has been proven that the mode superposition method is inefficient for large *Dofs* problem. For wave propagation problem, especially the thermal wave problem, the direct iteration on time like central difference time integration scheme is more efficient. Unluckily, a very large amount of *Dofs* should be considered to model and simulate the thermal wave problem due to the wide band of excitation and the tiny relaxion time. The total number of *Dof* usually achieves 10,000 for a reasonable response and avoiding the numerical oscillation, and the matrixes are all larger than 10,000 × 10,000. The central difference time integration scheme avoids the calculation of inverse matrix, however, “out of memory” still threatens the calculation. In addition, the large *Dofs* used in modelling to capture the high frequency wave characteristics further compounds “out of memory” problem due to the least stable time interval, which is determined by the Von Neumann conditioning number of the inspected model. For above reasons, we selected the central difference time integration scheme, however, with a special solving methodology to address the “out of memory problem” for title issue. It should be emphasized 10,000 even larger is not problem for some commercial software like ANSYS. We take this example here to say that direct calculation of inverse of a very large matrix is impossible in practice. To calculate the inverse of a large matrix is time-consuming, which in further compounded by the iteration on time.

(1) For the CV model. To solve Equation (32) via the central difference time integration scheme, the main difficulty focuses on the decomposition of large matrix to avoid the direct calculation like KΘ on the global matrix level. Assume the equations as described in Equations (40) and (41) according to the central difference time integration scheme:

(40) $$\frac{\partial \Theta_{\lambda }}{\partial \lambda }=\frac{\Theta_{\lambda +\Delta \lambda }-\Theta_{\lambda -\Delta \lambda }}{2\Delta \lambda }$$

(41) $$\frac{\partial^{2}\Theta_{\lambda }}{\partial \lambda^{2}}=\frac{\Theta_{\lambda +\Delta \lambda }-2\Theta_{\lambda }+\Theta_{\lambda -\Delta \lambda }}{\Delta \lambda^{2}}$$

Substituting Equations (40) and (41) for the corresponding derivatives in Equation (32):

(42) $$M\frac{\Theta_{\lambda +\Delta \lambda }-2\Theta_{\lambda }+\Theta_{\lambda -\Delta \lambda }}{\Delta \lambda^{2}}+C\frac{\Theta_{\lambda +\Delta \lambda }-\Theta_{\lambda -\Delta \lambda }}{2\Delta \lambda }+K\Theta_{\lambda }=G_{\lambda }$$

where Δλ is the step between the neighbor integration slice in time domain. To address the “out of memory problem”, Equation (42) is further rewritten as Equation (43) for the CV model:

(43) $$\underset{M^{0}}{\underset{︸}{\left(\frac{1}{\Delta \lambda^{2}}M+\frac{1}{2\Delta \lambda }C\right)}}\Theta_{\lambda +\Delta \lambda }=G_{\lambda }-\underset{\hat{F}}{\underset{︸}{K\Theta_{\lambda }}}+\underset{M^{1}}{\underset{︸}{\left(\frac{2}{\Delta \lambda^{2}}M\right)}}\Theta_{\lambda }-\underset{M^{2}}{\underset{︸}{\left(\frac{1}{\Delta \lambda^{2}}M-\frac{1}{2\Delta \lambda }C\right)}}\Theta_{\lambda -\Delta \lambda }$$

Note matrixes **M** and **C** are in a diagonal form, the inverse of matrixes **M**^0^, **M**^1^, and **M**^2^ can be easily obtained following the example:

(44) $${\left(M^{0}\right)}^{-1}=diag\left(\frac{1}{M_{ii}^{0}}\right)$$

Matrixes **M**^0^, **M**^1^, and **M**^2^ are stored in vector forms due to their diagonal property. Thereafter, we now focus on the component $\hat{F}$, which will be decomposed into the elemental level for calculation to avoid global multiplication. The specific algorithm is described in Table 1 referring to [46].

In the above algorithm, calculations are mainly conducted on the elemental level, and thus the “out of memory problem” is addressed.

(2) For the DPL model. The difficulty of solving Equation (38) based on the algorithm presented in Table 1 is generated by the non-diagonal property of matrix **H**. To address this issue, we proposed the following predict-update format. According to the central difference time integration scheme, Equation (38) is rewritten as:

(45) $$\underset{M^{0}}{\underset{︸}{\left(\frac{1}{\Delta \lambda^{2}}M+\frac{1}{2\Delta \lambda }C+\frac{\lambda_{T}}{2\Delta \lambda }K\right)}}\Theta_{\lambda +\Delta \lambda }=G_{\lambda }-\underset{\hat{F}}{\underset{︸}{K\Theta_{\lambda }}}+\underset{M^{1}}{\underset{︸}{\left(\frac{2}{\Delta \lambda^{2}}M\right)}}\Theta_{\lambda }-\underset{M^{2}}{\underset{︸}{\left(\frac{1}{\Delta \lambda^{2}}M-\frac{1}{2\Delta \lambda }C-\frac{\lambda_{T}}{2\Delta \lambda }K\right)}}\Theta_{\lambda -\Delta \lambda }$$

where the matrix **H** is replaced by $\lambda_{T}K$. Due to the non-diagonal property of **K**, the inverse of matrix **M**^0^ cannot be obtained following the example presented in Equation (43). To address this problem, we rewrite the non-diagonal part to the right of equation as:

(46) $$\underset{M^{0}}{\underset{︸}{\left(\frac{1}{\Delta \lambda^{2}}M+\frac{1}{2\Delta \lambda }C\right)}}\Theta_{\lambda +\Delta \lambda }=G_{\lambda }-\underset{\hat{F}}{\underset{︸}{K\Theta_{\lambda }}}+\frac{\lambda_{T}}{2\Delta \lambda }K\Theta_{\lambda -\Delta \lambda }-\frac{\lambda_{T}}{2\Delta \lambda }K\Theta_{\lambda +\Delta \lambda }+\underset{M^{1}}{\underset{︸}{\left(\frac{2}{\Delta \lambda^{2}}M\right)}}\Theta_{\lambda }-\underset{M^{2}}{\underset{︸}{\left(\frac{1}{\Delta \lambda^{2}}M-\frac{1}{2\Delta \lambda }C\right)}}\Theta_{\lambda -\Delta \lambda }$$

Thereafter, the inverse of $M^{0}$ can be efficiently computed. However, the appearance of the variable to be calculated, namely $\Theta_{\lambda +\Delta \lambda }$, makes it impossible for solving. In the predict-update iteration method, the value of the $\Theta_{\lambda +\Delta \lambda }$ on the right-hand side is assigned to be $\Theta_{\lambda }$ in the predicting phase:

(47) $$\Theta_{\lambda +\Delta \lambda }⇐\Theta_{\lambda }$$

Equation (46) is modified as (the predicting phase):

(48) $$\underset{M^{0}}{\underset{︸}{\left(\frac{1}{\Delta \lambda^{2}}M+\frac{1}{2\Delta \lambda }C\right)}}\Theta_{\lambda +\Delta \lambda }=G_{\lambda }-\underset{\hat{F}}{\underset{︸}{K\Theta_{\lambda }}}+\underset{M^{1}}{\underset{︸}{\left(\frac{2}{\Delta \lambda^{2}}M\right)}}\Theta_{\lambda }-\underset{M^{2}}{\underset{︸}{\left(\frac{1}{\Delta \lambda^{2}}M-\frac{1}{2\Delta \lambda }C\right)}}\Theta_{\lambda -\Delta \lambda }$$

(49) $$\left(\frac{1}{\Delta \lambda^{2}}M+\frac{1}{2\Delta \lambda }C\right)\Theta_{\lambda +\Delta \lambda }^{p}=G_{\lambda }-\left(1+\frac{\lambda_{T}}{2\Delta \lambda }\right)K\Theta_{\lambda }+\frac{\lambda_{T}}{2\Delta \lambda }K\Theta_{\lambda -\Delta \lambda }+\left(\frac{2}{\Delta \lambda^{2}}M\right)\Theta_{\lambda }-\left(\frac{1}{\Delta \lambda^{2}}M-\frac{1}{2\Delta \lambda }C\right)\Theta_{\lambda -\Delta \lambda }$$

The solving methodology of Equation (49) is similar with the algorithm shown in Table 1, and then one can get the predicted value $\Theta_{\lambda +\Delta \lambda }^{p}$. Substitute $\Theta_{\lambda +\Delta \lambda }^{p}$ for the $\Theta_{\lambda +\Delta \lambda }^{}$ on the right-hand side of Equation (49), the updating phase is obtained:

(50) $$\Theta_{\lambda +\Delta \lambda }^{}⇐\Theta_{\lambda +\Delta \lambda }^{p}$$

(51) $$\left(\frac{1}{\Delta \lambda^{2}}M+\frac{1}{2\Delta \lambda }C\right)\Theta_{\lambda +\Delta \lambda }^{u}=G_{\lambda }-K\Theta_{\lambda }+\frac{\lambda_{T}}{2\Delta \lambda }K\Theta_{\lambda -\Delta \lambda }-\frac{\lambda_{T}}{2\Delta \lambda }K\Theta_{\lambda +\Delta \lambda }^{p}+\left(\frac{2}{\Delta \lambda^{2}}M\right)\Theta_{\lambda }-\left(\frac{1}{\Delta \lambda^{2}}M-\frac{1}{2\Delta \lambda }C\right)\Theta_{\lambda -\Delta \lambda }$$

Solving Equation (51), the updated $T_{t+\Delta t}^{u}$ is obtained, and then substituted into Equation (46) for iteration until the convergence.

### 3.4. Definition of Boudary Condition and Initial Condition

Boundary condition and initial condition are important for thermal analysis. The thermal boundary conditions can be clustered into three types: (1) Dirichlet (the first type) boundary condition: the temperatures of some areas are given. (2) Neumann (the second type) boundary condition: the heat fluxes of some areas are given. (3) The mixture of (1) and (2) types. To implement the boundary condition on PDEs may be problematic for these quantities in parallel. However, one can define them in the above algorithms easily. Taking the first type and the second type boundary condition for example: (1) the first type boundary condition, in which temperature is fixed in special area. Let us pay attention to Table 1, in the last command in the loop, we calculated the temperature for time step 1, which is further used as the initial condition for time step 2. Therefore, one can restrain (i.e. set to be the given value) the corresponding temperature for restrained area to satisfy the first type boundary condition. The similar process is repeated in every time step and thus the boundary condition can be fulfilled. (2) the second type boundary condition, in which heat flux is fixed in special area. It is worth to point out that the accurate definition of the second type boundary condition in the present algorithm is impossible. It seems that we can implemented the first order derivative of temperature by Equation (40), by which the heat flux can be restricted. However, the heat flux in the non-Fourier problems, is no longer directly proportional to the temperature gradient, especially in heterogeneous materials or for low-temperature phenomena [47,48,49,50,51]. Of course, one may consider a higher order modification based on Equations (40) and (41), the first and the second order derivatives of temperature. But the method is still not accurate enough. For this reason, heat flux is usually treated as an independent state variable in calculations. Recently, an implicit scheme are presented by Reith and Kovács et al. [52], which successfully describes numerous beyond Fourier experimental findings. More details of the boundary problem can be referred to this work. The restrain of initial condition is to set the initial value to $\Theta_{-1}$ and $\Theta_{0}$, and thus the initial value of temperature and heat flux can be controlled.

### 3.5. Stability Conditions of Central Difference Time Integration

A disadvantage of the central differences method can be its conditional stability, which requires that the length of the time step Δλ be smaller than some critical value that is closely related to the dynamic properties of the discretized system:

(52) $$\Delta \lambda \leq \Delta \lambda_{critical}=\frac{2}{\omega_{n}}$$

where *ω_n_* is the shortest period of eigenvalue of the discrete system. The stable value of Δλ the finite element system is determined by the maximum of *ω_n_* in system. In practice, the value of Δλ is usually selected by trial-and-error method, the initial value of Δλ is set as:

(53) $$\Delta \lambda \leq \frac{\Delta s_{\min}}{c_{\max}}$$

where $\Delta s_{\min}$ is the minimum characteristic length of element. For one-dimensional case, it is the length of element, and it is the 2-norm (alternative) of all elemental edges for two-dimensional case. When the trial value induces an unstable solution, reduce the Δλ and repeat the calculation.

## 4. Numerical Results and Discussions

Utilizing the CV/DPL models and the corresponding solving formats, numerical computations are performed to display the lagging thermal behavior in various media under pulse-laser heating in the form defined in above sections. Mentioned that the situation $\lambda_{T}=0$ degenerates the DPL into the CV model, the CV model is deemed as a special case of the DPL model and thus different models are not emphasized in the analysis. In the following parts, discussions are organized by the following logic:

(1) Validate the convergence and accuracy of the presented WFEM method by comparing with the time domain spectral finite element method (SFEM) proposed by Ostachowicz and Kudela et al. [53], one of the best methods for the dynamic analysis, and the classical FEM. The comparisons about convergence and accuracy are conducted on one-dimensional structures. These methods are all coded by Matlab in the similar program structure. It should be mentioned that although the time consumption can be obtained by “tic, toc” in Matlab and the similar program structure are used, we do not compare the efficiency by time, however, by DOFs used.
(2) Different behaviors of the inspected systems are performed using the developed model, containing the wavy behavior ($\lambda_{T}=0$), the wavelike behavior ($\lambda_{T}=0.1$), the diffusive behavior ($\lambda_{T}=0.5$), and the over-diffusive behavior ($\lambda_{T}=1.5$). On this aspect, the applicability of the proposed model in different situations can be verified.
(3) Considering the simplicity of one-dimensional grids, the flexibility and applicability of the presented method are validated by comparisons on two-dimensional grids.

The boundary conditions of the following cases are all defined as the Neumann boundary heat flux is 0. For one-dimensional case, the boundary is set on the two tips of region. In two-dimensional case, the Neumann boundary is restrained on the boundary of region. For all cases, the 0 initial condition is applied, namely temperature change and the heat flux are all 0 at beginning. The physical parameters before dimensionless process are given for possible comparison: (1) the material with the thermal parameters *α* = 0.2301 × 10^-4^ m^2^/s, *τ*_0_ = 0.1720 ps, (2) geometrical parameters, *x* = 5 nm (equivalent to 1.257 in dimensionless domain) for one-dimensional case, radius *r* = 6 nm (equivalent to 1.508 in dimensionless domain) for two-dimensional case. 3) excitation parameter, *τ_p_* = 100 fs (equivalent to $\lambda_{p}=0.2907$), the reflectivity of irradiated surface is simply assumed as *R* = 0. 4) Temporal parameter, for one-dimensional case, *t* = 3 ps (equivalent to λ=8.7208), divided into 10,000 time steps; for two-dimensional case *t* = 1.5 ps (equivalent to λ=4.3604), divided into 10,000 time steps.

### 4.1. Convergence and Accuracy

**Figure 2:**
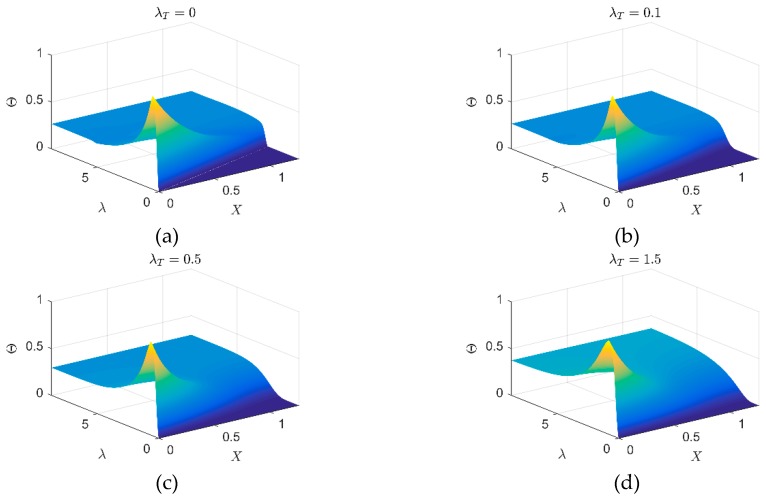
Thermal wave propagation in one-dimensional media: (**a**) $\lambda_{T}=0$; (**b**) $\lambda_{T}=0.1$; (**c**) $\lambda_{T}=0.5$; (**d**) $\lambda_{T}=1.5$.

**Figure 3:**
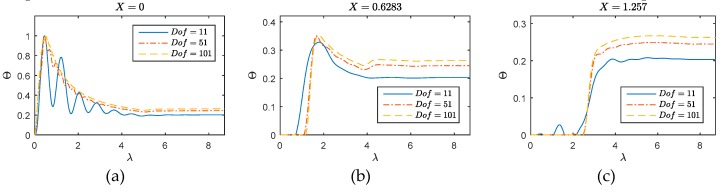
Performance and convergency of finite element method (FEM) on one-dimensional issue (**a**) *X* = 0; (**b**) *X* = 0.6283; (**c**) *X* = 1.257.

**Figure 4:**
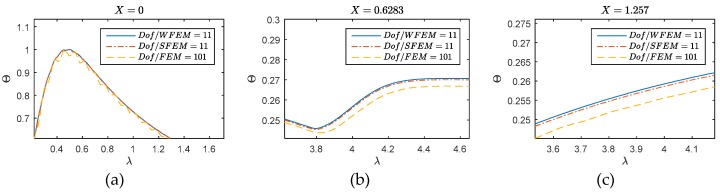
Comparisons on accuracy and efficiency among FEM, spectral finite element method (SFEM), and wavelet finite element (WFEM): (**a**) *X* = 0; (**b**) *X* = 0.6283; (**c**) *X* = 1.257.

**Figure 5:**
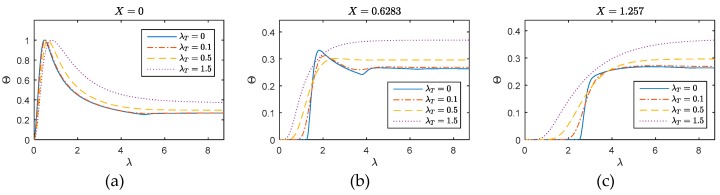
WFEM performances for different $\lambda_{T}$: (**a**) *X* = 0; (**b**) *X* = 0.6283; (**c**) *X* = 1.257.

The performances of the presented method on convergence and accuracy are validated by the comparison with SFEM and FEM on one-dimensional problem. As the dimensionless parameters are used, they are not emphasized here. The overview of the WFEM performance on one-dimensional thermal wave behavior can be found in Figure 2, where the thermal behavior on the total field are presented for different $\lambda_{T}$. However, it is observed that the differences contained in the sub-figures among different $\lambda_{T}$ is not evident. Thus, Figure 3, Figure 4 and Figure 5 are further presented for comparison. In this numerical example, the heat source is implemented on *X* = 0. The total length of the inspected region is 1.257. For different cases, the WFEM shows a good applicability. Covered by the strong thermal impulse, the differences induced by the variation of $\lambda_{T}$ are not clear on the interval X⊂[0,0.5]. However, on the interval *X* > 0.5, the dimensionless temperature change becomes smoother with the increase of $\lambda_{T}$, which means the diffusive behavior becomes more dominant and the wave behavior becomes weaker.

A detailed comparison on accuracy and efficiency is given in Figure 3 based on the same numerical scenario $\lambda_{T}=0$ used above. The compared methodologies are FEM, the classical method, SFEM, one of the best methods for dynamic analysis, and the presented WFEM. The FEM is conducted using the classical elements with *Dof* = 11, 51, and 101, and the compared SFEM and WFEM methods are conducted with the *Dof* = 11. The temperature changes on excited point (*X* = 0), middle of the media (*X* = 0.6283) and the tip of one-dimensional region (*X* = 1.257) are calculated by above methods. In Figure 3, the poor convergency of FEM is totally performed on this issue. The calculations based on *Dof* = 11 of FEM show the obvious numerical oscillation on different positions. At point *X* = 0 (left picture of Figure 3), the numerical oscillation of FEM results has essentially changed the real waveform. The dense grid can suppress this phenomenon, the FEM result of *Dof* = 101 is still beset by the oscillation (seen in the left picture in Figure 4). At point *X* = 0.6283 (middle picture of Figure 3), the wave behaviors are not clearly performed by FEM. Either the behavior the direct wave (near λ=2), or the arrival time of the primary reflection from the tip (near λ=4) cannot be accurately described by the FEM. At point *X* = 1.257 (right picture of Figure 3), even a false waveform can be observed near λ=1.5 in the responses obtained by FEM. Thus, the classical FEM cannot qualify the analysis of the title problem. For this reason, the FEM results for two-dimensional cases are not further presented.

Likewise, the WFEM (*Dof* = 11) and SFEM (*Dof* = 11) are introduced into analysis. The comparisons on accuracy and efficiency among FEM, SFEM and WFEM are given in Figure 4, where the zoom-out views on some key time intervals are presented for comparison. We can see the SFEM and WFEM achieve the higher accuracy with less *Dof*s used on the three inspected positions compared with FEM. The agreement between WFEM and SFEM further validates the effectiveness of the developed method. The comparison given in Figure 4 proves the WFEM to be an alternative methodology for thermal wave analysis.

The conclusion given by Figure 4, however, is not complete as the applicability of WFEM on $\lambda_{T}$ is not investigated yet. Thereafter, results in Figure 5 are supplied for this reason. Generally, the WFEM performs well for the inspected cases, no numerical oscillation can be observed. At point *X* = 0 (left picture of Figure 5), the arrivals of the peak move to the right and the primary reflection near λ=5 is blurred with the increase of $\lambda_{T}$. The diffusive behavior becomes more dominant. The phenomenon and tendency can be clearly observed in the responses on *X* = 0.6283 and 1.257. On the middle point (middle picture of Figure 5), responses for $\lambda_{T}=0$ present to be a typical wave behavior similar with the elastic wave, and the arrival time of the wave is instinct near λ=1.25. For diffusive ($\lambda_{T}=0.5$) and over-diffusive cases ($\lambda_{T}=1.5$), the arrival time cannot be defined as the thermal behavior is not wave-like. Compared the responses at *X* = 0.6283 with *X* = 1.257 (right picture of Figure 5), we can see the temperature disturbance arrives nearly the same time for these two positions, this suggests that in the diffusive and over-diffusive cases, the wave speed is nearly infinite.

### 4.2. Flexibility and Applicability

**Figure 6:**
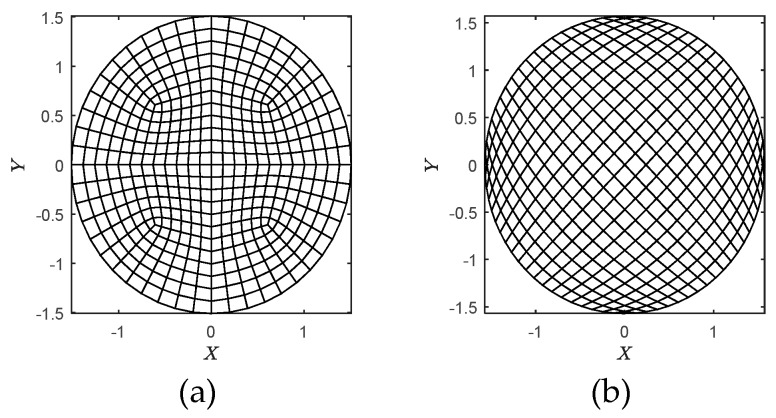
Mesh grids used in calculations: (**a**) grid A; (**b**) grid B.

The convergence and accuracy of the presented method have been verified in the above section. Hereafter, the flexibility and applicability of the presented method are validated by computations and comparisons on two-dimensional grids as shown in Figure 6. The inspected region with the dimensionless radius *r* = 1.508 is meshed by 432 and 400 WFEM elements as shown in Figure 6a,b, respectively. The heat source locates at point (−1.508, 0), namely the left tip of the region. Although the two cases are meshed in totally different fashions, the similar numbers of elements (432 and 400) are used in calculations to keep the comparability of simulated results.

**Figure 7:**
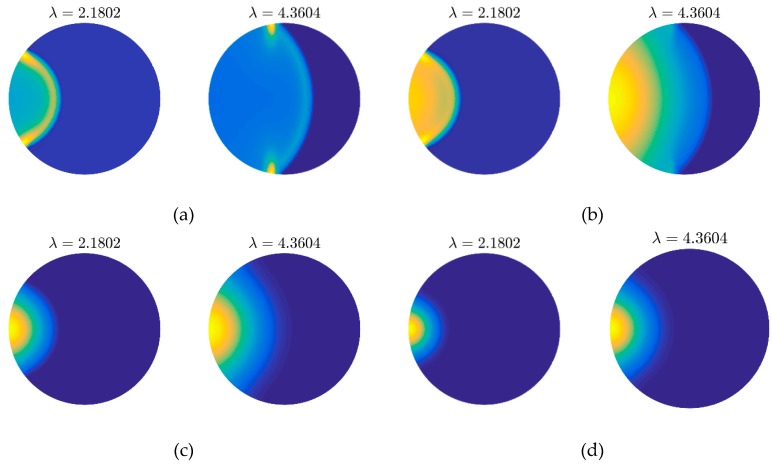
Thermal behaviors (snapshots) obtained by mesh grid in Figure 6a with different $\lambda_{T}$: (**a**) wavy behavior $\lambda_{T}=0$; (**b**) wavelike behavior $\lambda_{T}=0.1$; (**c**) diffusive behavior $\lambda_{T}=0.5$; (**d**) over diffusive behavior $\lambda_{T}=1.5$.

**Figure 8:**
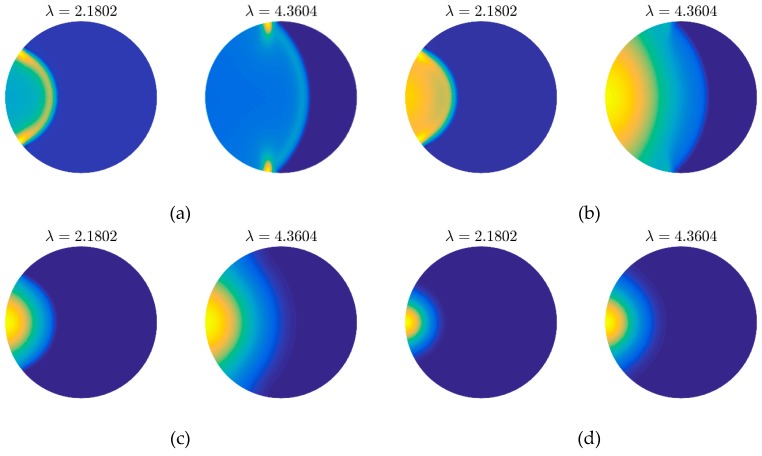
Thermal behaviors (snapshots) obtained by mesh grid in Figure 6b with different $\lambda_{T}$: (**a**) wavy behavior $\lambda_{T}=0$; (**b**) wavelike behavior $\lambda_{T}=0.1$; (**c**) diffusive behavior $\lambda_{T}=0.5$; (**d**) over diffusive behavior $\lambda_{T}=1.5$.

Firstly, the results obtained by the grids shown in Figure 6a,b are presented in Figure 7 and Figure 8 for comparison. It is very clear that we can get the same thermal behaviors via both two kinds of meshes. On another aspect, the quality of grid in Figure 6b is worse than that in Figure 6a since the heterogeneity, especially on the left and right ends. The result of which, however, achieves a similar accuracy with the fine mesh. This can verify the flexibility and applicability of the presented method to some extent.

Then, we will pay attention to the inner comparison of Figure 7, which illustrates the different thermal behaviors in the inspected area. Figure 7a presents the wavy behavior of temperature distribution changes in the inspected area. In this case, the pulsed thermal disturbance propagates in the form of wave. In the snapshot λ=2.1802, we can observe a clear circle wave front, on which the energy of the pulsed thermal disturbance is mainly focused. With the increase of dimensionless time (λ=4.3604), the energy is slowly absorbed by the media as the result of matrix **C** in the DPL model.

With the increase of $\lambda_{T}$ from 0 to 0.1, the sharp wave fronts in Figure 7a are smoothed and the portions of the disturbance are dissipated. Thus, we can see the influenced area of Figure 7b is larger than that of Figure 7a. Due to the increase of $\lambda_{T}$, matrix **H** plays the more important role in the damping term. As the consequence, the temperature changes on area after wave front becomes more evident compared with the wavy behavior. This is called the wavelike behavior because the concepts like wave front, reflection still can be used to describe the behavior. In both the wavy or wavelike behaviors, some hot points are the reflections of thermal wave to be focused, like the tips of wave fronts. When $\lambda_{T}=0.5$ (Figure 7c), all wavy features disappear, the disturbance caused by pulse transports by diffusion completely. The hot points appear on the tips of wave front are smoothed and the temperature peak in this case is always the heating spot (−1.508, 0). If we continue to enlarge $\lambda_{T}$ to 1.5, the over-diffusive behavior can be observed (as shown in Figure 7d). Compared with the normal diffusion, the larger $\lambda_{T}$ produces the higher diffusion rate in an early stage. However, a longer time is required to reach the thermal equilibrium.

**Figure 9:**
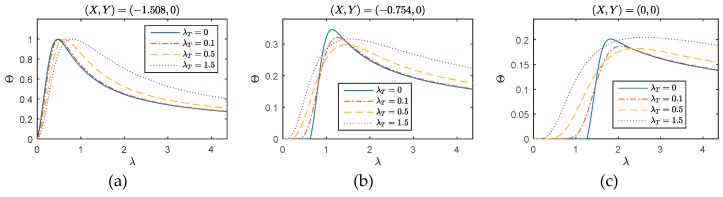
WFEM performances for different $\lambda_{T}$: (**a**) (*X*, *Y*) = (−1.508, 0); (**b**) (*X*, *Y*) = (−0.754, 0); (**c**) (*X*, *Y*) = (0, 0).

To illustrate and compare the thermal behavior shown in Figure 7 more clearly, the thermal responses on points (−1.508, 0), (−0.754, 0), and (0, 0) with different $\lambda_{T}$ are presented in Figure 9. Totally, the changing trends of the responses in two-dimensional media are similar with that in one-dimensional media, however, with longer time to achieve thermal equilibrium. In addition, the temperature change is also smaller compare with that in the one-dimensional case.

## 5. Conclusions

In order to describe the thermal behavior of film material, a new WFEM formulation for thermal behaviors in one- and two-dimensional media has been proposed in this work. The hyperbolic heat conduction model has been solved using the central difference scheme on time and wavelet interpolation in space. The proposed algorithm has been tested by comparison with the classical FEM and SFEM on the aspects of accuracy and efficiency. The flexibility and applicability for different mesh grids are validated in a two-dimensional case. The current work provides an alternative tool for the analysis of thermal analysis. It should be mentioned that the presented method cannot apply the heat flux boundary condition accurately due to the FEM formulation used in this work. It is worth to investigate the state variable based WFEM further to address this problem.

## Figures and Tables

**Table 1:** The main algorithm.

Main Algorithm
#1 Loop over elements e Calculate the elemental matrices **K**^e^, **M**^e^, and **C**^e^ Assemble matrices **M**, **C**, vector **G** Store every elemental stiffness matrix **K** #1 End of loop over element e Calculate the auxiliary vectors $M_{}^{0}$, $M_{}^{1}$, and $M_{}^{2}$ Apply the initial condition #2 Loop over time instants λ #21 Loop over elements e Load the stiffness matrix **K**^e^ Calculate ${\hat{f}}^{e}=K^{e}\Theta_{\lambda }^{e}$ on elemental level Assemble vector $\hat{F}$ by ${\hat{f}}^{e}$ #21 End of loop over elements e Calculate effective vector $\tilde{R}=G_{\lambda }-\hat{F}+M^{1}\Theta_{\lambda }-M^{2}\Theta_{\lambda -\Delta \lambda }$ Calculate $\Theta_{\lambda +\Delta \lambda }^{}={\left(M^{0}\right)}^{-1}\tilde{R}$ #2 End of loop over time instants λ.
